# The time-course linkage between hemolysis, redox, and metabolic parameters during red blood cell storage with or without uric acid and ascorbic acid supplementation

**DOI:** 10.3389/fragi.2023.1161565

**Published:** 2023-03-21

**Authors:** Alkmini T. Anastasiadi, Konstantinos Stamoulis, Effie G. Papageorgiou, Veronica Lelli, Sara Rinalducci, Issidora S. Papassideri, Anastasios G. Kriebardis, Marianna H. Antonelou, Vassilis L. Tzounakas

**Affiliations:** ^1^ Department of Biology, School of Science, National and Kapodistrian University of Athens (NKUA), Athens, Greece; ^2^ Hellenic National Blood Transfusion Centre, Acharnes, Greece; ^3^ Laboratory of Reliability and Quality Control in Laboratory Hematology (HemQcR), Department of Biomedical Sciences, School of Health and Welfare Sciences, University of West Attica (UniWA), Egaleo, Greece; ^4^ Department of Ecological and Biological Sciences, University of Tuscia, Viterbo, Italy; ^5^ Department of Biochemistry, School of Medicine, University of Patras, Patras, Greece

**Keywords:** red blood cell, glutathione, oxidative stress, uric acid, ascorbic acid, storage lesion, accelerated aging

## Abstract

Oxidative phenomena are considered to lie at the root of the accelerated senescence observed in red blood cells (RBCs) stored under standard blood bank conditions. It was recently shown that the addition of uric (UA) and/or ascorbic acid (AA) to the preservative medium beneficially impacts the storability features of RBCs related to the handling of pro-oxidant triggers. This study constitutes the next step, aiming to examine the links between hemolysis, redox, and metabolic parameters in control and supplemented RBC units of different storage times. For this purpose, a paired correlation analysis of physiological and metabolism parameters was performed between early, middle, and late storage in each subgroup. Strong and repeated correlations were observed throughout storage in most hemolysis parameters, as well as in reactive oxygen species (ROS) and lipid peroxidation, suggesting that these features constitute donor-signatures, unaffected by the diverse storage solutions. Moreover, during storage, a general “dialogue” was observed between parameters of the same category (e.g., cell fragilities and hemolysis or lipid peroxidation and ROS), highlighting their interdependence. In all groups, extracellular antioxidant capacity, proteasomal activity, and glutathione precursors of preceding time points anticorrelated with oxidative stress lesions of upcoming ones. In the case of supplemented units, factors responsible for glutathione synthesis varied proportionally to the levels of glutathione itself. The current findings support that UA and AA addition reroutes the metabolism to induce glutathione production, and additionally provide mechanistic insight and footing to examine novel storage optimization strategies.

## 1 Introduction

Stored red blood cells (RBCs) endure structural and functional/metabolic deteriorations, collectively known as storage lesion, that are mainly attributed to metabolic decline and redox imbalance and have been found to be linked to the transfusion outcome (Yoshida et al., 2019; Rogers et al., 2021). Some lesions concern typical RBC aging indicators, such as oxidative defects, altered cation homeostasis, and spheroechinocytosis (Antonelou et al., 2010; D'Alessandro and Zolla, 2013). At the same time, since the RBC unit represents a closed system, the metabolic and oxidative stresses are intensified during storage (D'Alessandro et al., 2015; Bardyn et al., 2018). For this reason, it is considered that stored RBCs undergo an accelerated senescence process, while the conventional, calendar way of measuring storage age does not seem to reflect their real physiological age (D'Alessandro et al., 2019; Koch et al., 2019).

Recent studies have shown “heritability” in some erythrocytic features during storage. Genome-wide association studies (GWAS) identified several loci associated with hemolysis (Page et al., 2021; Fang et al., 2022) or metabolism (Moore et al., 2022) of stored RBCs, and similar conclusions also arose from classic twin studies (van 't Erve et al., 2014a; van 't Erve et al., 2014b). Correlation analyses support these findings (Reisz et al., 2017; Tzounakas et al., 2021), and enrich them with inter-correlations, namely, correlations between distinct parameters, either before/during storage, or between different time points of storage (D'Alessandro et al., 2021; Thomas et al., 2021; Anastasiadi et al., 2022). Such information has the potential to indicate or hint at how variation in one parameter might affect another, and therefore, lead to a better understanding of the “dialogue” between discrete aspects of RBC life during storage. For instance, Oh *et al.*, have shown that the levels of nitrite oxidation during early storage can partly “predict” the release of free hemoglobin (Hb) and heme later on (Oh et al., 2015). Along the same lines, it has been suggested that hypoxanthine varies proportionally to hemolysis and echinocytosis (D'Alessandro et al., 2018).

Optimization of storage conditions and extension of RBC shelf-life is still in the spotlight of blood transfusion research. Antioxidant enhancement to deal with the elevated oxidative stress during storage is a common effort to achieve this goal (Amen et al., 2017; Barzegar et al., 2022; Nemkov et al., 2022). Uric (UA) and ascorbic acid (AA) are efficient natural antioxidants, the effect of which upon stored RBCs has been studied both distinctly (Dawson et al., 1980; Raval et al., 2013; Sanford et al., 2017; Tzounakas et al., 2022) and in combination (Bardyn et al., 2020; Tzounakas et al., 2022). A recent study by our team has shown that the addition of the two antioxidants ultimately mitigates oxidative lesions in RBCs, as exemplified by reduced oxidative lysis and membrane oxidation, and induces glutathione (GSH) synthesis through metabolic rerouting (Tzounakas et al., 2022). nonetheless, it has not been yet shown how the supplementation affects intra- and inter-associations between the levels of physiological and metabolic parameters throughout storage. For this purpose, this study aimed to examine the statistical links between early-, middle- and late-stored RBC features in units supplemented with UA and/or AA in comparison to untreated samples.

## 2 Materials and methods

### 2.1 Biological samples

The present study reflects a secondary correlation analysis of data derived from a previously reported RBC unit supplementation protocol (Tzounakas et al., 2022), which focused on between-group differences in physiological and metabolic parameters. In that study, thirty-four leukoreduced RBC units from healthy individuals containing citrate-phosphate-dextrose (CPD)/saline-adenine-glucose-mannitol (SAGM) were split under aseptic conditions into four subunits of equal volume. One subunit from every quartet was used as control, while the other three were supplemented with UA (in-bag concentration: 7–8 mg/dL), AA (in-bag concentration: 2.3 mg/dL), or their mixture. All units were stored for 42 days at 4°C and sampling was aseptically performed during early (day 7), middle (day 21), and late (day 42) storage. The study was approved by the Ethics Committee of the Department of Biology, School of Science, NKUA. Investigations were carried out upon donor consent, by the principles of the Declaration of Helsinki.

### 2.2 Physiology, redox, and metabolism parameters

As previously stated (Tzounakas et al., 2022), measurement of hemolysis (spontaneous, osmotic, mechanical, and oxidative lysis), intracellular reactive oxygen species (ROS), membrane lipid peroxidation and extracellular antioxidant capacity were performed in all samples. A selection of six samples per category provided metabolomic, proteasomal, and immunodetection data.

Briefly, regarding hemolysis parameters, spontaneously released Hb was measured in the supernatant using Harboe’s method, while the three induced-lysis measurements were spectrophotometrically performed post exposure to (a) descending NaCl concentrations, (b) mechanical stress using stainless steel beads (Raval et al., 2010) and (c) phenylhydrazine (PHZ; 17 mM). The levels of intracellular ROS were fluorometrically assessed using the redox-sensitive probe 5-(and-6)-chloromethyl-2′,7′-dichloro-dihydrofluoresceindiacetate, acetyl ester (CM-H_2_DCFDA) with and without external stimuli (tert-butyl hydroperoxide–tBHP; 100 μM). Malondialdehyde was detected at 532 nm post formation of a chromogenic complex with thiobarbituric acid (Tzounakas et al., 2022), whereas extracellular antioxidant capacity (total, UA-dependent, and UA-independent) was evaluated *via* the ferric reducing antioxidant power (FRAP) assay (Benzie and Strain, 1996).

After membrane and cytosol isolation by hypotonic lysis, the two fragments were subjected to incubation with fluorogenic proteasome substrates to measure the three proteasomal activities (chymotrypsin (CH)-, trypsin (TR)- and caspase (CASP)-like) *via* fluorometry (Anastasiadi et al., 2021). The membranes were also immunoblotted for a variety of membrane-binding proteins. Metabolomic analysis was performed in RBCs using an ultra-high-performance liquid chromatography (UHPLC) system coupled with a mass spectrometer (MS), as previously extensively described (Tzounakas et al., 2022).

### 2.3 Statistical analysis

**FIGURE 1:**
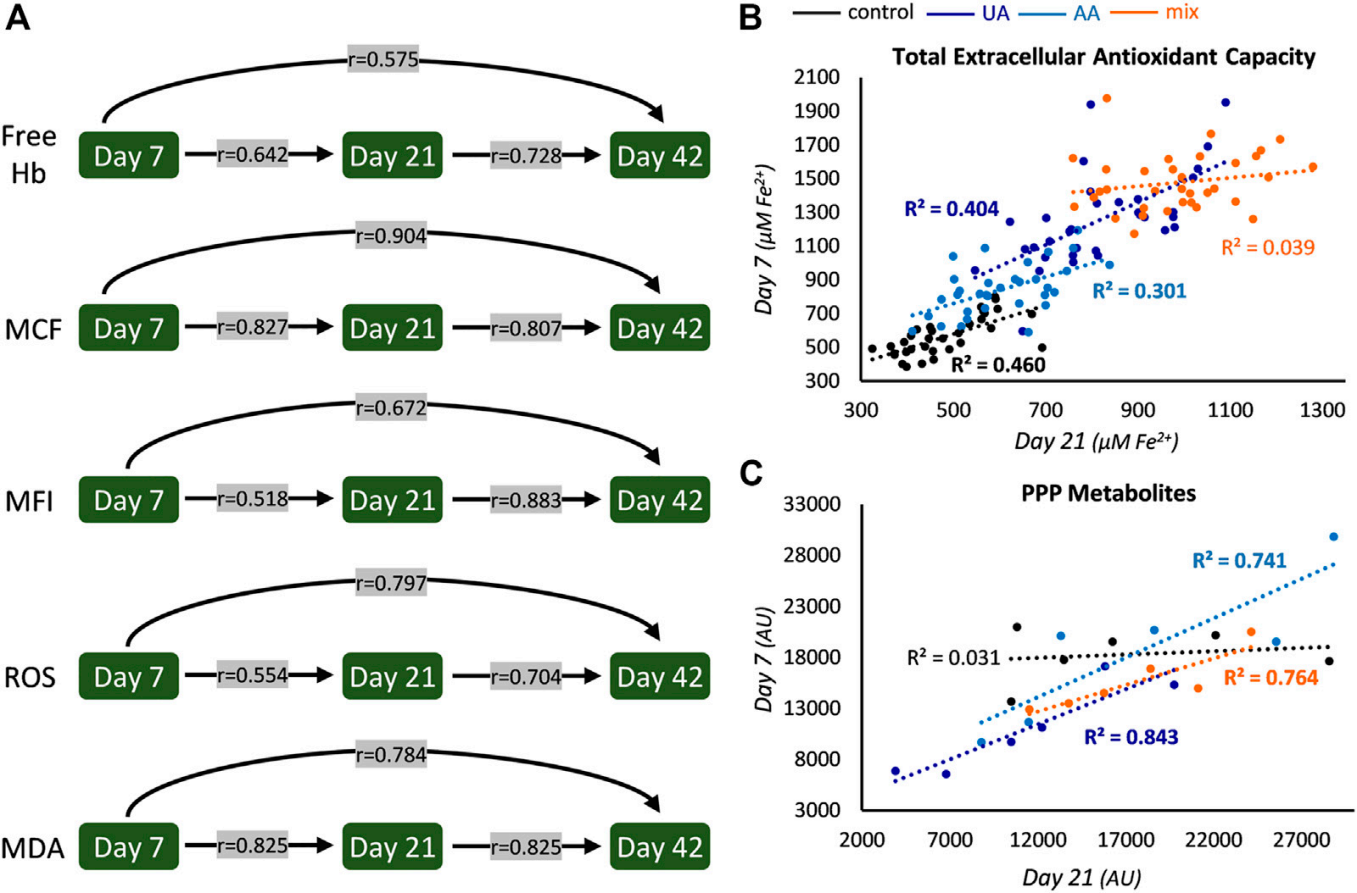
Intra-parameter correlations during storage. **(A)** Correlations between the levels of hemolysis and oxidative parameters at early, middle and late storage. All r values are statistically significant and concern controls, but similar statistically significant values were found in uric acid (UA)-, ascorbic acid (AA)- and mix-enhanced units (see also Figure 2). **(B)** Scatter plot of the levels of early and middle storage for extracellular antioxidant capacity (bold font *p* < 0.05). The same pattern with slightly different *R* ^2^ values was evident for all storage periods examined. **(C)** Scatter plot regarding the levels of pentose phosphate pathway (PPP) metabolites (for control and UA: D-erythrose 4-phosphate; for AA, sedoheptulose 7-phosphate; for mix: glucose 6-phosphate) between early and middle storage. Hb, hemoglobin; MCF, mean corpuscular fragility; MFI, mechanical fragility index; ROS, reactive oxygen species; MDA, malondialdehyde; AU, arbitrary units.

**FIGURE 2:**
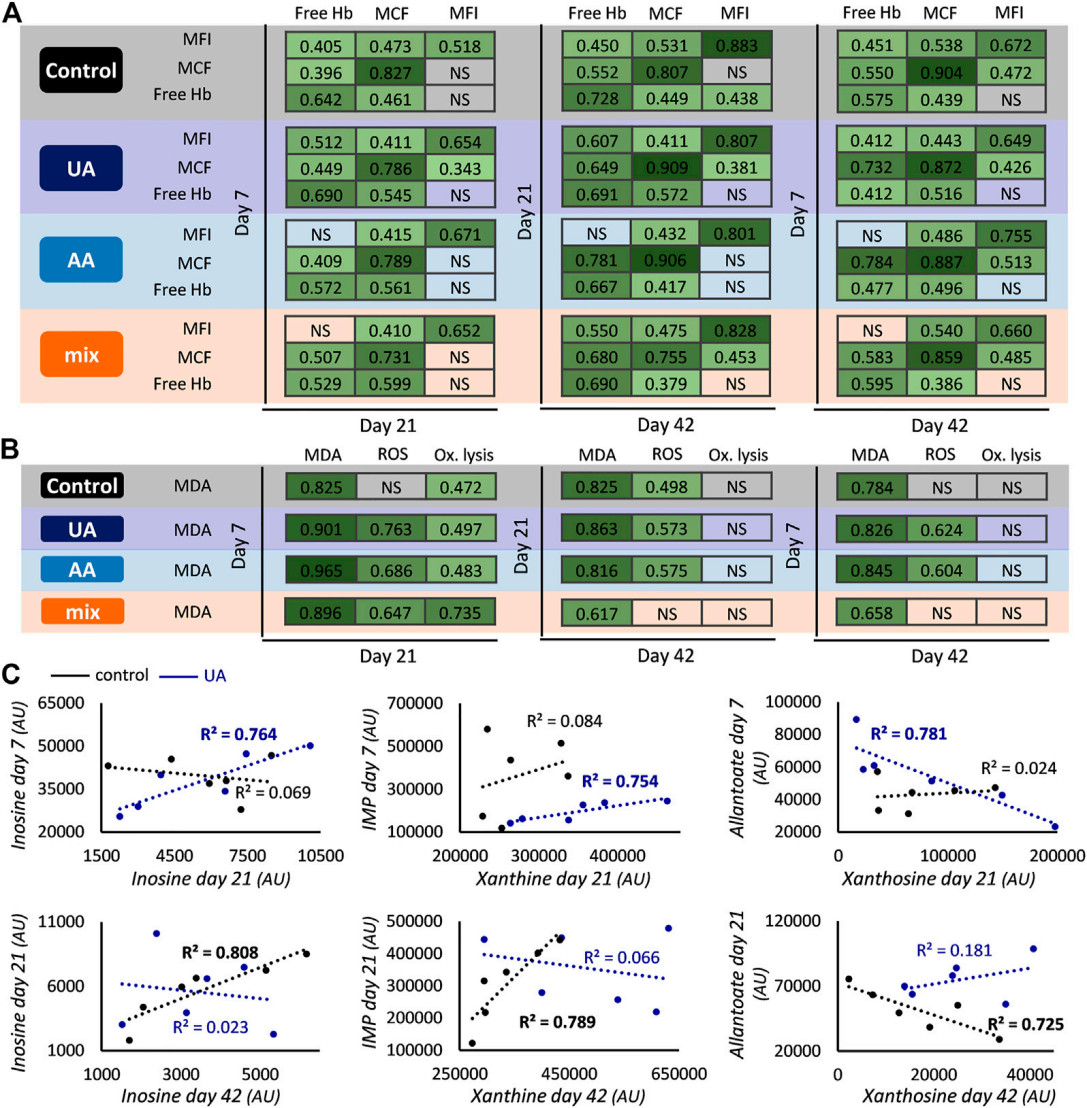
Inter- and intra-parameter correlations during storage. Correlations between the levels of hemolysis **(A)** and oxidative **(B)** parameters at early, middle and late storage for control, uric acid (UA)-, ascorbic acid (AA)- and mix-enhanced units. All r values shown exceed the threshold for statistical significance (*p* < 0.05). Scatter plots presenting correlations between the levels of early and middle or middle and late storage **(C)** regarding purine metabolites for control and UA-supplemented units (*R* ^2^ values with bold font: *p* < 0.05). Xanthosine day 21 control values were ten-fold multiplied to fit the graphical representation. Hb, hemoglobin; MCF, mean corpuscular fragility; MFI, mechanical fragility index; NS, not significant; Ox, oxidative; MDA, malondialdehyde; ROS, reactive oxygen species; AU, arbitrary units.

For statistical analysis, SPSS (Version 26.0, IBM Hellas, Athens, Greece, administered by NKUA and the University of Patras) computer software was used. All variables were tested for normal distribution profile and the presence of outliers (Shapiro-Wilk, Kolmogorov–Smirnov tests and detrended normal Q-Q plots as appropriate) and then Pearson’s and Spearman’s tests were performed to assess correlations between parameters. Since Pearson’s test is sensitive to outliers, such values were excluded, and the analysis was performed again to minimize false positive correlation results. If the outcome of the analysis was not modified, the outlier was included back in the group. In the case of intra-parameter correlations or parameters belonging to the same category (hemolysis, redox, and purine oxidation variables of Figures 1, 2) and presenting interconnections with each other, a Bonferroni-like adjustment was applied to increase the reliability of multiple comparisons. The metabolomics and physiological data used originate from a previous work of the antioxidant supplementation project (Tzounakas et al., 2022). *p* < 0.05 was considered statistically significant.

## 3 Results

### 3.1 Intra-parameter correlations

In all four groups, the levels of spontaneous, osmotically-induced, and mechanically-induced lysis, along with the levels of intrinsic intracellular ROS and membrane lipid peroxidation of any preceding storage time point were found directly proportional to the levels of every upcoming one (Figure 1A). Especially regarding hemolysis parameters, which are of great clinical importance for transfusion medicine, the possibility to (partly) “predict” end-of-storage levels based on early in-bag measurements might prove to be crucial. In Figure 1A the r values shown correspond to the control units, but similar correlations, with slightly different r values, arose in all supplementations (e.g., r values of ROS, day 7 vs. day 21: 0.667, 0.700, and 0.430 for UA-, AA- and mix-supplemented units, respectively; *p* < 0.05).

On the other hand, some intra-parameter correlations were affected in part on in all of the modified units. For example, in control units the levels of total extracellular antioxidant capacity of earlier time points (namely, 7 and 21) were found positively correlated to the ones of advanced time points (namely, 21 and 42); this was maintained in UA- and AA-enhanced units but it was “lost” in mix supplementations (Figure 1B). In the same context, intra-correlations were observed in all supplementations between the early and middle levels of distinct parameters of glycolysis (e.g., in UA, glyceraldehyde 3-phosphate dehydrogenase: *r* = 0.938; in AA, glycerate 3-phosphate: *r* = 0.829; in mix, fructose 1,6-bisphosphate: *r* = 0.943; *p* < 0.05) and the pentose phosphate pathway (PPP; Figure 1C), a finding that was absent in control samples. Of note, oxidative hemolysis presented correlation only between its early and middle levels and only in control samples (*r* = 0.504, *p* < 0.05). All these intra-parameter correlations point towards the dominance of a donor-signature, that is either universal (in all conditions tested) or revealed under specific storage solutions.

### 3.2 Inter-parameter correlations between factors of the same category

A general (namely, present in all four subgroups) crosstalk was observed between the values of spontaneous hemolysis and the two cellular fragilities, suggesting that RBCs prone to hemolysis (with/without external stimuli) remain as such from early to later storage. The addition of AA affected the link between the mechanical fragility of preceding periods and hemolysis of advanced time points (Figure 2A). Another differentiation in this crosstalk concerns the correlation between early/middle osmotic fragility and middle/late mechanical fragility, which is mainly evident in units containing UA (Figure 2A). Inter-correlations were also found regarding classic parameters of oxidative stress. Lipid peroxidation of early storage presented positive links with ROS accumulation (in all enhancements) and oxidative lysis (in all groups) of the middle time point (Figure 2B). In the case of oxidative lysis, this link was completely lost in late storage values, while the pattern was sporadically maintained regarding ROS accumulation (Figure 2B). Based on these data, it seems that relative physiological parameters may exhibit similar variation profiles during storage.

The addition of UA, a member of purine metabolism, additionally affected inter-parameter correlations in the purine metabolic pathway. While the end-storage levels of purine metabolites varied proportionally to those of day 21 in controls, this pattern was “moved” to earlier time points in UA-enhanced units, resulting in correlations between purines of day 7 and day 21 (Figure 2C). It should be noted that while most of these inter-correlations were positive, the levels between xanthosine and metabolites downstream of uric acid were negatively correlated (e.g., allantoate/xanthosine in Figure 2C). This profile was completely absent from AA- and mix-treated blood units.

### 3.3 Inter-parameter correlations between factors of distinct categories evident in all groups

**FIGURE 3:**
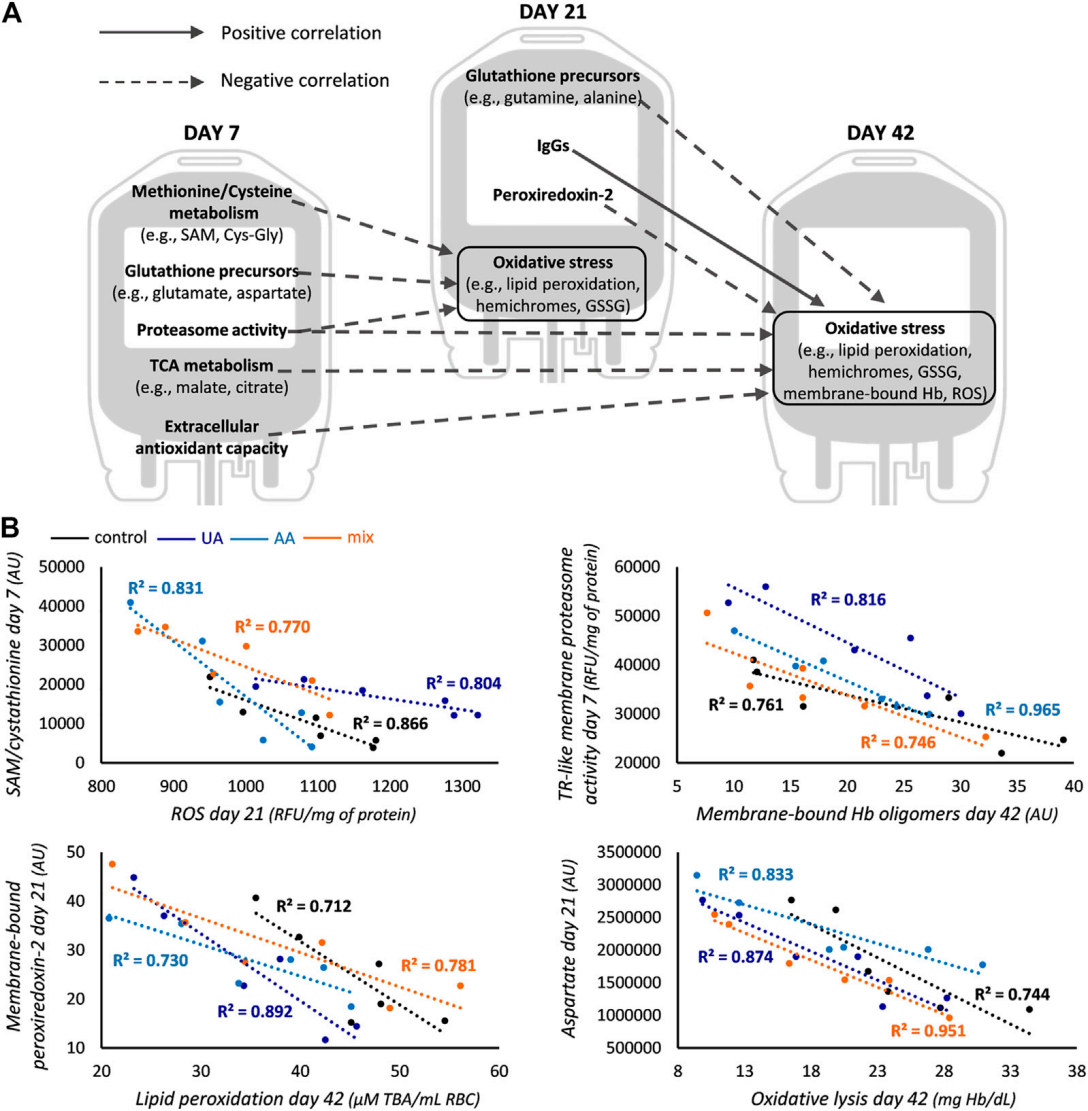
Correlations common across all groups. **(A)** Correlations between redox, proteostasis and metabolism parameters of early and middle storage with oxidative stress of middle and late storage for control, uric acid (UA)-, ascorbic acid (AA)- and mix-enhanced units. **(B)** Representative scatter plots regarding some of the correlations graphically presented in panel **(A)**. In the first scatter plot S-adenosyl methionine (SAM) is shown for controls and cystathionine for supplementations. All *R* ^2^ values shown exceed the threshold for statistical significance (*p* < 0.05). TCA, tricarboxylic acid; GSSG, glutathione disulfide; Hb, hemoglobin; ROS, reactive oxygen species; AU, arbitrary units; RFU, relative fluorescence units; TR-like, trypsin-like; TBA, thiobarbituric acid.

Oxidative stress is one of the main causes of storage lesions; for this reason, all possible manifestations of oxidative stress, such as insults to the membrane, intracellular ROS accumulation, relative metabolic markers, or oxidative lysis, were put in focus to unravel their possible links to other physiological variables of the RBC units. The antioxidant enhancement left several correlations between (mainly) metabolites/antioxidants and oxidative stress unaffected compared to the control (Figure 3A). The rationale followed was to consider correlations involving parameters of the same category as common between the four groups. For example, when metabolites of tricarboxylic acid (TCA) metabolism were linked to markers of oxidative stress in controls, and other metabolites of the same pathway were also linked to oxidative stress markers in supplementations, this correlation was considered as common between supplemented and control units.

Early-storage GSH precursors (namely, methionine-cysteine metabolism and synthesis *via* amino acids) were found inversely correlated to oxidative stress markers of middle storage in all subgroups (Figure 3B). This was also extended between their middle and advanced storage levels. The proteasomal activity of early-storage was also negatively linked to oxidative stress markers of upcoming time points, as in the case of membranic TR-like activity and Hb oligomers (Figure 3B) or cytosolic CASP-like activity and ROS (day 7 vs. day 21: −0.840, −0.815, −0.822 and −0.897, r values for controls, UA-, AA- and mix-supplemented units, respectively; *p* < 0.05). Interestingly, the abundance of membrane-bound peroxiredoxin-2 (prdx2) in day 21 RBCs was also inversely correlated to oxidative stress factors of late storage, as exemplified by the case of lipid peroxidation (Figure 3B). All these common correlations point towards the effective action of antioxidant and proteostasis potential upon oxidative lesions, highlighting their functional interplay. The only common positive correlation was observed between the membrane levels of immunoglobulins (IgGs) in middle storage, with the membrane levels of protein oxidation and Hb in late storage (e.g., for controls and mix-treated units: membrane-bound IgGs day 21—membrane carbonylation day 42 r = 0.865, r = 0.825, respectively; for UA- and AA-treated units: membrane-bound IgGs day 21—membrane-bound Hb day 42 r = 0.894, r = 0.841, respectively; *p* < 0.05).

### 3.4 Unique inter-parameter correlations between factors of distinct categories

**FIGURE 4:**
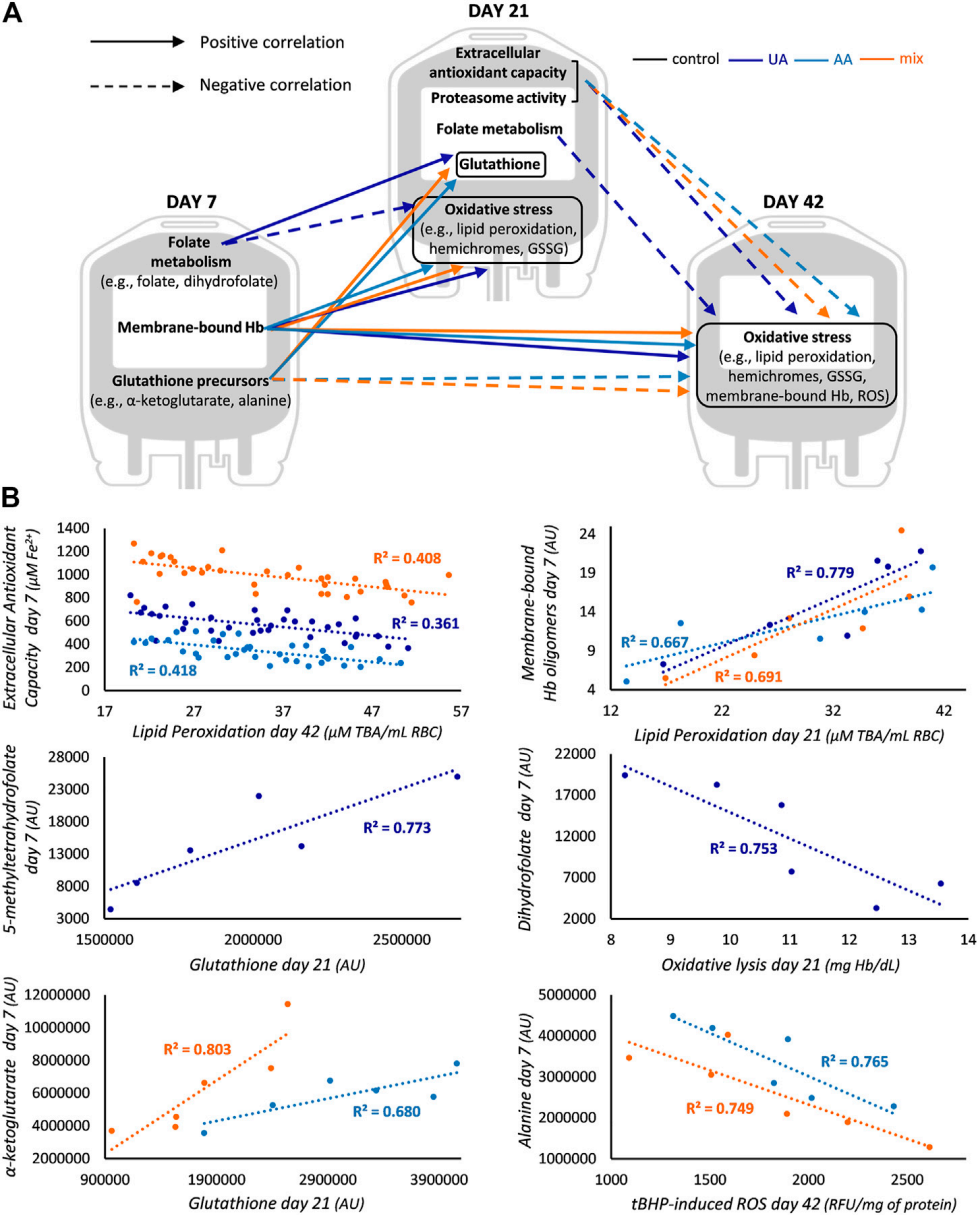
Differential correlation profile in enhanced blood units. **(A)** Unique correlations between redox, proteostasis and metabolism parameters of early and middle storage with redox parameters of middle and late storage for uric acid (UA)-, ascorbic acid (AA)- and mix-enhanced units. **(B)** Representative scatter plots regarding some of the correlations graphically presented in panel **(A)**. In the first scatter plot total antioxidant capacity (AC) is shown for mix, UA-dependent AC for UA and UA-independent AC for AA units. All *R* ^2^ values shown exceed the threshold for statistical significance (*p* < 0.05). Hb, hemoglobin; GSSG, glutathione disulfide; ROS, reactive oxygen species; TBA, thiobarbituric acid; AU, arbitrary units; tBHP, tert-butyl hydroperoxide; RFU, relative fluorescence units.

Apart from the abovementioned common correlations, some associations were only evident in part on in all supplementations (Figure 4A). The levels of membrane-bound Hb or Hb oligomers during early storage varied proportionally to those of oxidative stress markers of either middle or late storage in all supplementations (Figure 4B). Additionally, extracellular antioxidant capacity (and more specifically, total for mix-, UA-dependent for UA- and UA-independent for AA-units) of day 21 presented an inverse correlation with oxidation markers of late-storage RBCs. The same target was also negatively associated with the proteasomal activity of middle-stored RBCs (e.g., CASP-like cytosolic activity day 21—oxidative lysis day 42: *r* = −0.829, *r* = −0.886 and *r* = −0.841 for UA-, AA- and mix-supplementations, respectively; *p* < 0.05), forming (when combined with data from Figure 3) a complete correlation pattern between preceding and upcoming time points in all supplementations.

The early levels of metabolites of folate cycle were proportional to the middle levels of GSH in UA-enhanced RBCs, while the levels of classical GSH precursors (e.g., α-ketoglutarate) were positively linked to the same middle-storage target in the other two supplementations (Figure 4B). Moreover, in UA-modified units, early and middle storage folate metabolites (e.g., dihydrofolate) were inversely linked to middle and late storage oxidative markers, respectively, while in AA- and mix-enhanced units, the early levels of GSH precursors (e.g., alanine) were negatively related to late storage oxidative stress (Figure 4B). In this way, and considering the abovementioned common correlations, in the two supplementations that contained AA, GSH synthesis metabolites of preceding time points anticorrelated with oxidative stress markers of upcoming ones.

## 4 Discussion

Several studies provide evidence regarding the dialogue between distinct metabolic or physiological parameters of RBCs during storage. Such information hints at interrelations among storage phenotypes and sets the basis for targeted experimentation to better understand and optimize preservation in the blood bag. After the addition of UA and/or AA, key factors of storage performance, such as antioxidant defenses and oxidative defects, presented an improved profile (Tzounakas et al., 2022). The present study supports that the time-course correlation between the previously reported storage parameters remained in some cases unaffected by antioxidant enhancement (e.g., hemolysis features) or, in other cases, exhibited unique patterns only in supplemented units (e.g., glutathione synthesis or membrane-bound Hb).

### 4.1 Hemolysis and redox parameters as donor-signatures in stored RBCs

The maintenance of proportional storage, osmotic and mechanical hemolysis levels, among all time points in every condition tested, suggests that hemolysis is more a function of donor features than of additive solution modifications. According to previous studies, the osmotic fragility of stored RBCs is strongly donor-dependent since it varies proportionally to the circulation levels (Tzounakas et al., 2021), being moderately associated with genes implicated in RBC structure and volume (Page et al., 2021). The undeniable contribution of structural proteins and ion channels to mechanical fragility (Smith et al., 2018; Svetina et al., 2019), leads to the assumption that there is a corresponding donor effect. In the same context, twin studies support that genetic factors contribute substantially to spontaneous hemolysis during storage (Van 't Erve et al., 2015). Overall, in the current study, despite the multiparametric nature of storage hemolysis (Van 't Erve et al., 2015; Reisz et al., 2017), the donor effect prevails inside the blood unit throughout storage.

The proportional storage motif of intracellular ROS accumulation and membrane lipid peroxidation is a finding upon which our research team stumbles for the first time, but our previous correlation works mostly focused on pre-storage–storage linkages. Therefore, the new environment, which is dissimilar to the *in vivo* in terms of redox equilibrium, might be bringing this specific donor-signature to the surface. Interestingly, a recent GWAS revealed a panel of metabolites strongly associated to the variation in genes implicated to RBC redox status, including the repair of oxidatively-damaged lipids (Moore et al., 2022). The fact that the extracellular antioxidant capacity −previously shown to be proportional to the pre-storage levels (Reisz et al., 2017)− failed to maintain the correlation pattern in the mix-treatments, might be attributed to the excessive enhancement, which led to a “burnout” effect when both antioxidants were added (Tzounakas et al., 2022).

Regarding metabolic aspects, classic twin studies and GWAS have shown a genetic control of glycolysis and PPP in stored RBCs (van 't Erve et al., 2014b; Weisenhorn et al., 2016; Moore et al., 2022). In our cohort, this donor-signature is lost between the time points of storage in the untreated units. The detrimental effect of storage stresses on energy metabolism (Rogers et al., 2021) seems to prevent the appearance of such correlations. It is known that already by the first 2 weeks of storage, glucose metabolism is rerouted from glycolysis to PPP to counteract oxidative stress, while alterations in band 3 protein -a “switch” for glucose metabolic flows- hold back its regulatory role in stored RBCs (Messana et al., 2000; D'Alessandro et al., 2015; D'Alessandro et al., 2017b; Issaian et al., 2021). nonetheless, the levels of distinct glycolytic and phosphate pentose metabolites varied proportionally from early to middle storage in supplemented samples, suggesting the achievement of an energy-related metabolic rewiring, as previously reported (Bardyn et al., 2020).

### 4.2 Interconnections of same-category parameters: Hemolysis, oxidative stress, and purine metabolism

Several RBC interactome/network analyses in a variety of RBC samples, ranging from distinct genetic backgrounds to stored erythrocytes, point towards the presence of hub nodes of biologically relevant parameters (Goodman et al., 2007; Issaian et al., 2021; Sae-Lee et al., 2022). In a similar context, the currently presented data showed that all types of hemolysis (except for oxidative one) are related to each other, while erythrocytes of high (or low) lipid peroxidation at earlier time points show increased (or decreased) ROS accumulation and oxidative lysis later on, a finding in line with the previously reported link between lipid peroxidation and hemolysis in murine models (Howie et al., 2019).

The striking absence of oxidative hemolysis from both intra- and inter-parameter hemolysis correlations, as well as from redox links to its late storage levels, makes room for intriguing explanations. Firstly, it seems that lysis after oxidative stimuli “belongs” to a different branch of parameters (mostly redox-related) than the ones attributed to corpuscular phenomena, as supported by a recent GWAS (Page et al., 2021). In addition, having in mind that (a) until middle-storage RBCs age in a more “ordered” way, at least in terms of energy and redox metabolism (Bordbar et al., 2016; Paglia et al., 2016; D'Alessandro et al., 2017b), and (b) beyond this time point RBCs accumulate more and more defects (Bardyn et al., 2017; Francis et al., 2020; Barshtein et al., 2021), it seems plausible to support that oxidative lysis becomes even more multiparametric at late storage resting its strong interconnection with other variables (including storage hemolysis) (Kanias et al., 2017) difficult. Osmotic and mechanical fragilities are not usually found intercorrelated even though they seem to affect one another (Tan et al., 2010). The steady correlation profile between them in UA-units requires further examination, especially since a similar link has been reported in donors with higher UA-dependent antioxidant capacity (Anastasiadi et al., 2022).

The sole addition of UA fueled the intercorrelation of purine metabolites earlier during storage, while at the same time upregulating a significant part of their metabolism (Tzounakas et al., 2022). Such information provides the ability to monitor proportional changes in purine oxidation molecules with the potential to undermine post-transfusion RBC performance such as inosine and hypoxanthine (D'Alessandro et al., 2017a; Nemkov et al., 2018), while the inverse correlation between allantoate and xanthosine seems to support our previous hypothesis, that under these conditions, UA acts on xanthosine rather than on classic stress-biomarkers (hypoxanthine, allantoin) (Kand’ar and Zakova, 2008; Nemkov et al., 2018). Regarding heritability in purines, the storage levels of urate have been linked to the gene of folate receptor (Moore et al., 2022), a finding that seems to match the observed alteration of folate metabolites in UA-supplementations (Tzounakas et al., 2022).

### 4.3 GSH metabolism, antioxidants, and proteostasis anticorrelate with oxidation markers

In all groups, RBCs with the equipage to produce GSH seem to be resistant to oxidative defects in the following days of storage. GSH is a critical contributor to the RBC redox homeostasis both as a scavenger and as an enzymatic cofactor (Pompella et al., 2003; Galano and Alvarez-Idaboy, 2011), but its levels drop early during storage (Bordbar et al., 2016) impelling scientists to propose the addition of its precursors (or even GSH itself) in the blood units (Dumaswala et al., 2000; Whillier et al., 2011). The observed general correlations, combined with the elevated amounts of some GSH precursors and the diminished oxidative insults in modified units (Tzounakas et al., 2022), strengthen our theory regarding metabolic rerouting to produce GSH and thus, enhance the antioxidant defenses. This observation should be further evaluated, since tracing experiments have shown that stored RBCs present minimal GSH production (Whillier et al., 2011; D'Alessandro et al., 2017b). Nevertheless, the fact that folate intermediates (in UA units) and GSH amino acid precursors (in AA and mix units) of day 7 vary proportionally to middle-storage GSH further reinforces our hypothesis, especially since these distinct route alterations were considered responsible for the impressive GSH levels in the modified units of day 21 (Tzounakas et al., 2022). This is additionally supported by the inverse correlations between GSH precursors/folate metabolites and oxidative stress phenotypes in the supplemented units. It should not be omitted that the superior GSH levels could also be explained by its potentially reduced sequestration by Hb (Fenk et al., 2022) due to the antioxidant intervention.

Another general correlation regarding oxidative stress markers was observed with extracellular antioxidant capacity and proteasomal activities of early storage. Donors who possess increased extracellular antioxidant powers have erythrocytes characterized by improved storability profile (Tzounakas et al., 2015), therefore the first association was rather anticipated. On the other hand, proteasome dominates the reported RBC interactomes (Anastasiadi et al., 2021; Sae-Lee et al., 2022) and there are indications that in RBCs it is tasked with decongesting the cell from oxidated proteins and especially Hb (Neelam et al., 2011; Abi Habib et al., 2020). There lies the fact that membranes with increased proteasomal activity are characterized by decreased membrane-bound oxidized Hb later during storage. In modified units, the same correlation additionally existed between middle and late stored RBCs. We can only hypothesize that antioxidant enhancement effectively protected (a) proteins, reducing the levels of over-oxidized protein crosslinks that cannot be processed by the proteasome machinery (Delobel et al., 2012) and (b) the proteasome itself (Delobel et al., 2016), making it more capable of dealing with oxidative stress beyond middle storage. Correspondingly, the binding of the antioxidant prdx2 to the membrane seems to safeguard its components from oxidation (Bayer et al., 2016; Möller et al., 2023).

The link between membrane-bound IgGs and Hb-binding or membrane oxidation is interesting. IgGs become more abundant in RBC membranes during both *in vivo* (Magnani et al., 1988) and accelerated (Luten et al., 2004; Kriebardis et al., 2007) aging, due to the appearance of senescence antigens (Dinkla et al., 2012). One of the current theories regarding RBC aging supports that band 3 is “trapped” in a rare conformation *via* the binding of IgGs extracellularly and denatured Hb intracellularly, forming a neo-antigen (Badior and Casey, 2018). The observed linkage between membrane-bound antibodies and Hb is in line with this “molecular clock” theory. Only in the modified units, the membrane binding of Hb in early storage was related to oxidative phenomena in later time points. Having in mind that the enhanced blood units were characterized by decreased oxidative stress, this correlation might emerge only in low levels of oxidative lesions that can act as a statistical threshold. In any case, increased binding of denatured Hb to the membrane is a potent oxidation source, that contributes to protein defects, lipid peroxidation, and membrane/cytoskeleton integrity (Alayash, 2022).

## 5 Concluding remarks

The currently reported correlations reinforce the hypothesis that UA and AA supplementation reroutes the metabolism of stored RBCs to produce or maintain GSH and cope with the redox imbalance. Moreover, they provide supporting information regarding donor-signatures, including RBC fragility and intracellular ROS, the intra-association of which during storage is not affected by the different additive solutions, as well as hints about intervention targets to optimize storage conditions. A very promising example is proteasomal activity, the upregulation of which could differentiate the proteostasis network of stored RBCs to cope better with the oxidative insults in blood bank conditions. Whether such a hypothesis will prove to be fruitful remains to be determined by future studies.

## Data Availability

The data analyzed in this study is subject to the following licenses/restrictions: The currently reported results are a secondary analysis of previously published data. Datasets will be made available upon request from the authors. Requests to access these datasets should be directed to VT, vtzounakas@upatras.gr.
